# Antimicrobial Stewards Must Aim for Balance in “Going Beyond the 9 to 5”

**DOI:** 10.1093/ofid/ofaf717

**Published:** 2025-12-15

**Authors:** Rebekah W Moehring, Timothy P Gauthier

**Affiliations:** Department of Medicine, Infectious Diseases, Duke University, Durham, North Carolina, USA; Department of Medicine, Infectious Diseases, Duke Center for Antimicrobial Stewardship and Infection Prevention, Durham, North Carolina, USA; Baptist Health South Florida, Clinical Pharmacy Enterprise, Miami, Florida, USA

**Keywords:** antibiotic stewardship, antimicrobial stewardship, on call, work force

Congratulations to Brace et al. for publication of survey data from 69 United States (US) hospitals in this edition of OFID. This study describes dedicated personnel resource allocation and coverage hours for hospital antimicrobial stewardship programs (ASPs), which updates our understanding from the last major US survey of 244 hospitals by Doernberg et al. in 2018 [1]. Since then, ASPs have matured, survived a pandemic, adopted telehealth practices, and grown system-based programs that spread stewardship expertize across varied and remote settings [2, 3]. Summarized full-time equivalent (FTE) descriptions as presented here can allow stewardship leaders to learn from varied practice models to design what works best for their own institutions and patients. This survey's focus on gathering information for “on call” programs—defined as ASP team activities occurring outside of traditional work hours—is novel. Most responses were from large >500 bed, academic hospitals. Approximately a third (24/69) of surveyed hospitals reported on call activities, including 17/69 (25%) during nights, 22/69 (32%) during weekend days, and 20/69 (29%) during holidays. Nine (13%) reported compensation for on call activities. From these data, we can conclude that on call ASP models are not a standard expectation for inpatient ASPs, even among this sample of large US hospitals.

The need for well-designed and resourced ASPs exceeds the work force of infectious diseases (ID) specialists. Brace et al.'s survey of 69 US hospitals including 1 critical access hospital represents a small sample compared to the distribution of hospital-based care in the US. For example, in 2023 the NHSN surveyed 3394 general acute care hospitals and 1286 critical access hospitals [4]. In small hospital settings, dedicated stewardship FTEs are hard to obtain without strategies to share pooled resources or training and delegation of stewardship activities to existing clinical personnel [5, 6]. Attempting to additionally cover off-hours with a centralized stewardship team in such settings is hard to imagine when there is limited or no baseline of dedicated FTE for regular hours. One feasibility study of preauthorization showed that zero community hospital study sites could adopt strict implementation including after-hours coverage [7]. Thus, the finding that large programs with existing dedicated FTE were more likely to offer on call ASP services is no surprise. The most common activity reported by 18 of 24 (75%) hospitals with on call programs was answering ASP/ID questions. Though potentially impactful for individual patients, being available to answer ASP/ID questions at all hours likely has little impact on hospital antibiotic use if implemented without other active interventions. Stenehjem et al.'s landmark, cluster-randomized trial of small community hospitals utilizing centralized expertize provided a 24-hour ID “hotline” in all three models of ASPs tested. However, only hospitals randomized to the most intensive program with centralized experts performing daytime interventions via telemedicine reduced antibiotic use [8]. These findings suggested that 24-hour availability of ID expertize was not the key programmatic intervention.

While impacting antibiotic use is important, maintaining the well-being of the antimicrobial stewardship workforce is paramount for enabling long-term success and sustainability. Stewardship champions are faced with the insurmountable task of improving the safe and effective use of antimicrobials in all care areas. Prioritizing the work of the day to enable the greatest impact is a challenge when the number of patients to review outnumbers the minutes in the day. Low-value tasks and those that further moral injury or burnout must be mitigated or avoided. One 2021 survey suggested over one-third of ASP pharmacist and physician respondents met criteria for burnout, with higher rates among pharmacists [9, 10]. IDSA has highlighted the critical shortage of ID physicians, and dwindling match rates have ID fellowship programs searching for ways to keep trainees' well-being a high priority [11]. ASPs that traditionally staffed overnight preauthorization calls with ID fellows have since pivoted to morning reviews and prospective audit and feedback [12, 13]. Adding responsibilities that may increase staff stress and anxiety beyond normal business hours seems unwise in such an environment. Dr. Emily Heil described that antimicrobial stewards are not enthusiastic about offering unsolicited advice and being the “antibiotic police” of their facility [14]. Stewardship pharmacist and thought leader Dr. Debra Goff noted three common themes tied to attrition of stewardship pharmacists: lack of work-life balance, no dedicated time to conduct research, and not feeling appreciated for efforts to improve patient care [15]. Although on call services may be a source of pride and feasible for well-resourced ASPs, there is substantial risk for on call programs to disrupt work-life balance and negatively impact the well-being of stewardship personnel.

A major goal for ASPs is to empower primary prescribers to gain knowledge and incorporate stewardship principles into their daily practice, even when not directly intervened upon by the stewardship team. Being a steward is being a motivator, a coach, planting a seed and seeing it grow in colleagues' clinical reasoning patterns. If ASPs are positioned as a fee-for-service program that includes expectations for on call coverage, that role could change. In one-to-one stewardship interventions, we ask questions, provide anticipatory guidance, develop trust, and set up clinical rationales to make it easy to make the next responsible antibiotic decision. Offering advice “for your next patient…” to emphasize a learning point is what handshake rounds are made for, why we see indirect effects with persuasive stewardship strategies, and how we extend influence beyond an individual patient [16–18]. The risk of clinicians forgetting their responsibility for judicious prescribing because they rely on continuous ASP/ID availability and “turn off” their critical thinking could ultimately limit ASP effects. In contrast, seeing the slow progress of learning and behavior change in the clinicians we serve is both sustainable and satisfying.

So, when might ASP leaders consider adding on call activities to their program design? We provide a list of proposed benefits and possible risks of on call programs to consider (Table 1), and suggest a thoughtful, Plan-Do-Study-Act approach. First, establish and define clinical scenarios occurring during off-hours that could benefit from stewardship guidance. This might require review of calls or antibiotic decisions during off-hours to recognize gaps and to identify low-value activities. Second, prioritize needs based on patient safety and consider alternative strategies to address them. Third, develop a strategy that balances well-being with access to ID expertize when needed. For example, a tiered approach to preauthorization during overnight hours can allow first doses of restricted drugs with favorable safety profiles for subsequent morning reviews (eg, carbapenems) and reserve calls for more toxic agents where diagnostic reasoning from ID consultants or prompts for surgical consultation are urgent (eg, amphotericin). Fourth, make sure ASP and ID consultant teams discuss and agree that such risks require their input for off-hours scenarios and how to best communicate those rationales to front-line clinicians. Finally, no programmatic decision should be considered a “forever” plan. Reassess and learn with the aim of designing systems-based solutions for off-hours scenarios. Adjust ASP workflow and coverage as primary clinicians' stewardship-focused practice patterns improve, FTEs contract or expand, and ASP priorities evolve.

**Table 1.** Balancing the Potential Benefits and Risks of On-Call Antimicrobial Stewardship Program Services

Potential Benefits		Potential Risks	
Improved Compliance and Continuity of Care	Creates opportunity to guide adherence to restricted antibiotic policies and best practices outside normal business hours (eg, use of order sets, consulting specialists)	Staff burnout^a^	Increases risk of fatigue, reduced job satisfaction, and risk for program staff turnover. Requirement to be available in times of low-reception can creates anxiety and stress
Improved patient outcomes	In settings where continuity of care during off-hours is a major need, on-call ASP services may improve patient outcomes	Neutral patient outcomes	In settings where continuity of care during off-hours is not a major need, on-call services may not have an impact on patient outcomes
Increased program visibility	Requirements to interact with stewardship program team members increase contact points, may assist in relationship building	Accountability and ownership for antibiotic decisions	Can dilute prescriber ownership of antibiotic decisions if stewardship staff assume too much control. Can blur lines of which staff “own” a given care area
Targeted education	Provides real-time opportunities for provider education	Resistance to change, difficult conversations	In programs where the stewardship program is not well established, clinicians or departments may give pushback and complain of micromanagement. Reducing physician autonomy/increased barriers to having an order verified can negatively impact satisfaction. Tensions may arise when clinical preference differs—especially when program staff are not physically present and the ordering provider is. Difficult or low-value interactions may contribute to ASP burnout
Compensation opportunities	Creates potential for additional remuneration for stewardship staff	Work-life balance disruption	Erodes boundaries that support personal time and recovery for ASP staff
Mobile work opportunities	Can align with remote work arrangements	More device time and communication overload	Reduces the little device free time program staff may have had available to them. Increased after-hours communication volume and documentation demands
		Program cost	Involves equipment and operational expenses (eg, hardware, software, and staff compensation)
Enhanced data generation	Documentation and workload data may support program metrics and longevity	Administrative burden	Increases complexity for program management, may contribute to inequity in distribution of workload between staff
Early intervention	Enables timely input from knowledgeable specialists during critical decision-making moments, which can expedite improved antibiotic prescribing	Access delays	Can unintentionally slow antibiotic initiation or limit prescriber access to start treatment while awaiting responses during limited ASP staffing hours
		Liability risk	Making recommendations without full access to patient records or circumstances can bring medico-legal concerns or make it more difficult to ensure patient/care giver preferences are considered [19]. Documentation may not be consistent with standard care when off-site staffs are engaged. Privacy and data security risks may arise with potential to violate HIPAA or local policies
Trainee inclusion value	Offers learning opportunities for trainees and early career clinicians, potentially enhancing mentorship roles	Trainee supervision challenges	Trainees may lack experience or authority to make independent decisions and navigate organizational politics in high-stakes circumstances that arise, leading to inefficiencies, miscommunication, or avoidable turmoil
Innovation and research opportunity	May be possible to get grant funding or perform innovative research about on call duties	Reduced reserve for innovation and research	If overtasked, personnel may not have time to innovate or participate in research

^a^This potential risk must be given exceptional consideration above all other factors.

ASP leaders have the expertize to create systems that meet the needs of their patients and clinicians, yet preserve well-being, and create sustainable environments of continued, supportive personal growth. We encourage you to use the data presented here in *OFID* to reflect on your current approach, learn from your ID community, and further innovate on strategies that create both balance and impact.
